# Latent Profile of Internet and Internet Game Usage Among South Korean Adolescents During the COVID-19 Pandemic

**DOI:** 10.3389/fpsyt.2021.714301

**Published:** 2021-09-23

**Authors:** Dongil Kim, Junwon Lee, JeeEun Karin Nam

**Affiliations:** ^1^Department of Education, Seoul National University, Seoul, South Korea; ^2^Graduate School of Education, Ewha Womans University, Seoul, South Korea

**Keywords:** Internet gaming, adolescents, Internet addiction, South Korea, sex, mental health

## Abstract

**Introduction:** Globally, more people are spending time on the Internet and gaming since the outbreak of the Coronavirus Disease 2019 (COVID-19). Consequently, concerns about developing behavioral addiction of adolescents have been raised. Such risk could be greater for adolescents in South Korea where the majority of adolescents have access to the Internet and own a smartphone. In fact, statistics indicate that Korean youths are spending significantly more time on the Internet and gaming during the COVID-19 pandemic. Previous studies on the patterns of time spent on the Internet and Internet gaming show inconsistent results. The aim of this study is to investigate the latent profiles of the Internet and Internet game usage among adolescents in South Korea.

**Method:** Data from a national survey on elementary and middle school students across South Korea were used. The sample consists of 3,149 respondents, and 2,984 responses were analyzed after removing missing responses. Latent profile analysis was performed to investigate the number of latent profiles for the Internet and Internet game usage time. To validate the profiles, differences in problematic gaming behavior, sex, and neuroticism were examined.

**Results:** Seven profiles were found: Casual User, Moderate User, Smartphone User, Internet User, PC Internet Gamer, Heavy User, and Excessive User. Validation of the profiles indicated differences in problematic gaming behavior, sex, and neuroticism among selected profiles.

**Conclusion:** This study presented different profiles of the Internet and Internet game usage among adolescents in South Korea. Profiles with higher game usage time scored higher in problematic game use compared to other profiles. Males were more likely to be in the profiles with high gaming time, and females were more likely to be in Internet and Smartphone User profiles. The results indicate that Internet and Internet gaming usage patterns could be classified by the type of device used and the content of the Internet.

## Introduction

During the Coronavirus Disease 2019 (COVID-19) pandemic, social distancing, school closures, and quarantine became the new norm worldwide. These phenomena resulted in people spending more time at home and on the Internet (1). For example, Steam, the biggest digital personal computer (PC) gaming platform and market, announced that it has reached the highest number of concurrent users in its 16-year service history at 20,313,451 (2). As for mobile gaming, Google reported that the duration and frequency of gaming increased in two-thirds of mobile gamers across 10 countries (3). Online gaming increased by 75% in the USA (4).

With increased Internet and game usage due to the COVID-19 pandemic, there have been concerns regarding an elevated risk of developing behavioral addictions, especially Internet and gaming addiction (5–9). Increased levels of depression, anxiety, and stress due to the pandemic (10, 11) could make individuals more susceptible to addictive behaviors such as Internet surfing and gaming (6). In particular, children and adolescents could be more vulnerable to such risks (5, 12). Recent studies found a link between adolescents' mental health and their use of the Internet and gaming during the COVID-19 pandemic (13–15).

The risk of developing Internet addiction and Internet gaming addiction could be particularly high for the adolescents in South Korea. Among Korean adolescents, 99.1% use the Internet regularly, 58.8% play a PC game, and 82.4% play a mobile game (16). A significant percentage of these adolescents reported that their time spent on PC game (45.5%) and mobile game (56.6%) increased after the COVID-19 outbreak. Thus, an in-depth investigation into the trend of increased time spent on Internet and Internet gaming of South Korean adolescents is warranted.

### Addictive Behavior of Internet and Internet Gaming

Different diagnostic criteria for Internet addiction have been proposed under the terms such as “Internet addiction disorder,” “problematic Internet use,” “pathological Internet use,” and “Internet dependency” (17). Griffiths (18) suggested that Internet behaviors could be addictive much like substance addiction. This perspective brought Internet gaming disorder (IGD) in Section III of the 5th edition of Diagnostic and Statistical Manual of Mental Disorders (DSM-5) (19). In 2019, the 11th edition of the International Classification of Disease (ICD-11) also added a classification of GD. Despite the inclusion of IGD in DSM-5 and GD in ICD-11, the debate regarding the definition, diagnosis, and treatment of Internet and gaming addiction is still ongoing (20–26).

One issue is the distinction between Internet addiction disorder and IGD. Generally, Internet gaming-related addictive behaviors have been considered as a subtype of Internet addiction. Young (27) introduced the concept of Internet addiction with five subtypes—one of which was computer addiction with obsessive gaming. Young (28) later redefined Internet addiction as an impulse control disorder and specified excessive gaming as one of three subtypes of Internet addiction. In practice, however, the terms Internet addiction and IGD have been used interchangeably (29) and research studies have presented contradictory results. Tiego et al. (30) reported that the pattern of problematic Internet usage was represented by the level of overall severity rather than severity of a specific Internet activity. On the other hand, Király et al. (31) argued that problematic Internet use and problematic Internet gaming are different. Their results showed that problematic Internet use showed associations with all kinds of Internet activities while problematic Internet gaming was correlated with Internet gaming only. More recently, a systematic review by Burleigh et al. (32) suggested that Internet addiction and GD could be different constructs as they showed differently altered brain activities.

Another debate is about the difference between Internet gaming by PC and smartphone. In the past, IGD studies focused heavily on computer games (33). However, with the introduction of a smartphone and its global popularity, users of smartphone Internet games or mobile games increased dramatically. The total revenue for the mobile game industry was $86.3 billion in 2020, which increased seven times since 2012 (34). In 2020, 70.5% of South Koreans played at least one type of games, and 91.1% of gamers played mobile games (16). Compared to PC Internet games, mobile games have distinct characteristics such as accessibility and availability (35), as well as the purpose and the context of playing (36). While some scholars argue that mobile games have potential risks of social isolation and losing control similar to traditional videogames (36), results from related studies are inconsistent. In a study by Lopez-Fernandez et al. (37), one-third of participants used mobile games and using mobile games did not predict problematic usage. Also, Paik et al. (38) compared the prevalence of IGD among gamers by PC, smartphone, and both. The results indicated that gamers that used both media showed higher IGD prevalence and psychological symptoms while smartphone-only gamers had the lowest IGD prevalence. However, Liu et al. (39) presented that mobile gaming and the frequency of smartphone usage were significantly associated with smartphone addiction.

Whether time spent on the Internet and Internet gaming is a meaningful indicator of addictive behavior is also an important discussion. Although more time spent on the Internet and Internet gaming does not directly lead to addictive behaviors, the association between time spent and addictive behaviors using the Internet has been frequently reported. In the pathology model, scholars [(Chou and Hsiao, 2000; Chou et al., 1998; Hur, 2006; Morahan-Martin, 1999, (27) as cited in Tokunaga and Rains (40)] suggest that problematic Internet use is considered as a clinical pathology that psychological problem increases the time spent on the Internet which ultimately leads to problematic Internet usage (40). On the other hand, the deficient self-regulation perspective (41) suggests that amount of time spent is rather an outcome of problematic Internet usage (40). As these two models explained how problematic Internet usage is related to time spent on the Internet, the results from Tokunaga and Rains (40) showed that problematic Internet use significantly predicted time spent using the Internet. Similarly, in the study of Rho et al. (42), weekday game time was a risk factor for IGD, and in Laconi et al. (43), time spent on the Internet was a significant predictor of IGD score. Also, there was a strong correlation between time spent playing online games and IGD compared to offline games (44) and time spent on online gaming was associated with negative consequences (45). However, the results of more recent studies were inconclusive, with one study in China (46) finding a substantial link between IGD and daily gaming time, but another in South Korea finding only a limited relationship (47).

### The Present Study

At the face of an increased risk for developing addictive Internet and Internet gaming behavior worldwide, the present study investigated into the latent profiles of South Korean adolescents in terms of their time spent on the Internet by PC and smartphone and Internet gaming by PC and smartphone. The purpose of the present study is to explore if unique patterns of Internet media usage exist, and if those patterns differ depending on the type of media (Internet vs. game) and device used (PC vs. smartphone). The present study consists of two parts. First, a latent profile analysis of usage patterns will be performed based on the time spent on the Internet by PC and smartphone as well as the time spent on Internet gaming by PC and smartphone. Second, to validate the uniqueness of the identified latent profiles, differences in problematic game usage, sex, and neuroticism will be examined as predictors of profile membership, as differences among those factors have previously been reported (48–52).

## Methods

### Sample

The data used in this study were secondary data obtained from a national survey conducted by the National Youth Policy Institute. For the survey, elementary (grades 4–6) and middle school students (grades 7–9) from all seven provinces of South Korea were recruited using quota sampling based on the 2020 Statistical Yearbook of Education published by the Ministry of Education. The data were collected in person by a survey agency from November 2020 to December 2020. In total, 1,500 responses from elementary students and 1,649 responses from middle-school students were collected. There was no evidence of systematic missing. Also, because the number of missing responses was not significant, listwise deletion was used. After the deletion, a total of 2,984 responses were analyzed. The mean age of the participants was 13.6 years, and 48.1% of them were female.

### Measures

#### Time Spent on Internet and Internet Game

The time spent on four Internet-related activities was measured by self-reported open-ended questions: time spent on the Internet using PC, time spent on the Internet gaming using PC, time spent on smartphone except gaming, and time spent on Internet gaming using smartphone. The participants were asked to write down the average amount of time (in hours and minutes) spent on four activities during a weekday within the last 3 months. All the responses were converted into minutes. Although the participants were asked to answer using any possible natural numbers, the vast majority of responses were answered by an increment of 30 min. For the analysis, responses of 0 min were coded as 0, ≤ 30 min as 0.5, over 30 min and ≤ 60 min as 1, and so on.

#### Problematic Game Usage

Problematic game usage was measured by the Maladaptive Game Use Scale developed by the Korea Creative Content Agency (53). The scale measures the negative consequences of game usage within the past 1 year. The scale consists of 21 items measured on a four-point Likert scale, ranging from 1 (*strongly disagree*) to 4 (*strongly agree*). The scale has seven factors: withdrawal, tolerance, excessive usage, control impairment, compulsive usage, neglecting daily activity, and gaming despite negative consequences. Having a score of 9 or higher on three or more factors would be classified as “problematic usage.” The scale showed high internal consistency (Cronbach's α = 0.92) as well as acceptable construct validity, convergent validity, and concurrent validity (53). For the present study, the internal consistency was 0.94 for the total scale and 0.65 to 0.90 for the subscales.

#### Neuroticism

Neuroticism was measured using two items on a four-point Likert scale ranging from 1 (*strongly disagree*) to 4 (*strongly agree*). Two items inquired about the frequency of mood fluctuations and depression symptoms. Higher scores indicate higher neuroticism.

#### Data Analysis

Because there are no strong hypotheses about how people spend their time on the Internet, the aim of the present study was to identify unique latent groups (54). Thus, latent profile analysis (LPA) was performed to investigate the number of unique patterns on the Internet and Internet game usage time by PC and smartphone among adolescents in South Korea. To determine the optimum number of latent profiles, fit indices such as Akaike's information criterion (AIC), Bayesian information criterion, sample-size adjusted BIC (SABIC), entropy, and likelihood ratio tests such as Lo–Mendell–Rubin adjusted likelihood ratio test (LMR-LRT) and bootstrap likelihood ratio test (BLRT) were considered (55, 56). Also, parsimony, simplicity, clarity, and interpretability (56) were considered in identifying the number of profiles.

To validate the profiles, multinomial regression analysis and one-way analysis of covariance were performed. Multinomial analysis was conducted on sex and “problematic usage” to predict class membership using the R3STEP procedure (57). One-way analysis of covariance (ANCOVA) was performed after controlling for sex and school level to compare problematic game usage among profiles. Bonferroni correction was applied to adjust for comparing multiple profiles.

LPA and multinomial regression were conducted using Mplus 8.2 software, and one-way ANCOVA was conducted using SPSS 22.0 software.

## Results

### Descriptive Statistics and Correlation Analysis

Mean, standard deviation, and correlation analyses were performed, and the results are presented in Tables 1, 2. Adolescents spent an average of 2.16 h per day on their smartphones and 1.08 h per day on PC Internet games. Being a male correlated positively with the total MGUS score and time spent playing Internet games on a PC and negatively with neuroticism and smartphone usage time. Neuroticism showed a significant correlation with the total MGUS score, time spent on the Internet by PC, mobile gaming by smartphone, and smartphone use.

**Table 1:** Descriptive statistics of usage time and problematic game usage.

	**Mean**	**Standard deviation**
Internet game by PC	1.08 h	1.52
Internet by PC	1.51 h	1.64
Mobile game by smartphone	1.76 h	1.72
Smartphone	2.16 h	2.17
MGUS total	32.62	10.48

**Table 2:** Correlation between MGUS total score, usage time, neuroticism and sex.

	**Gender**	**MGUS total**	**Neuroticism**	**Internet game by PC**	**Internet by PC**	**Mobile game by smartphone**	**Smartphone**
Gender	1						
MGUS total	0.219^**^	1					
Neuroticism	−0.164^**^	0.127^**^	1				
Internet game by PC	0.305^**^	0.337^**^	0.003	1			
Internet by PC	0.011	0.122^**^	0.096^**^	0.291^**^	1		
Mobile game by smartphone	−0.023	0.244^**^	0.096^**^	0.176^**^	0.169^**^	1	
Smartphone	−0.154^**^	0.043^*^	0.158^**^	0.110^**^	0.278^**^	0.204^**^	1

* *p < 0.05*,

** *p < 0.01*.

### LPA

LPA was performed to determine if heterogeneous profiles of Internet and Internet game usage time by different devices exist among adolescents in South Korea. Based on previous studies about the possible subtypes of Internet and Internet game addiction, six to seven classes were expected to emerge. To determine the number of profiles, fit indices such as AIC, BIC, SABIC, entropy, and likelihood ratio tests such as LMR-LRT and BLRT were compared from unitary solution until the likelihood ratio tests were not significant. The LMR-LRT was not significant after nine profiles but AIC, BIC, SABIC, and BLRT reported improved model fit after nine profiles (see Table 3). The entropy for all nine profiles was above 0.8, ranging from 0.87 to 0.90.

**Table 3:** Fit indexes of latent profile analysis on Internet and Internet game usage time.

**Number of profiles**	**AIC**	**BIC**	**SABIC**	**Entropy**	**LMR-LRT, *p*-value**	**BLRT, *p*-value**
2	57,934.77	58,012.78	57,971.48	0.895	<0.001	<0.001
3	57,205.8	57,313.82	57,256.63	0.888	<0.001	<0.001
4	56,515.01	56,653.03	56,579.95	0.885	<0.001	<0.001
5	56,365.35	56,533.38	56,444.42	0.865	0.0156	<0.001
6	55,906.43	56,104.47	55,999.61	0.863	<0.001	<0.001
7	55,498.83	55,726.87	55,606.13	0.876	0.0030	<0.001
8	55,023.70	55,281.74	55,145.11	0.883	0.0033	<0.001
9	54,841.95	55,130.00	54,977.48	0.868	0.479	<0.001

*AIC, Akaike information criteria; BIC, Bayesian information criteria; SABIC, sample size adjusted information criteria; LMR LRT, Lo–Mendell–Rubin adjusted likelihood ratio test; BLRT, bootstrap likelihood ratio test*.

After the analysis of fit indices, theoretical and conceptual considerations were taken into account when deciding on the number of profiles. Specifically, six, seven, and eight solution models were compared for parsimony and interpretability. In the eight-solution model, despite the best statistical fit, no meaningful distinction could be made between class 2 and class 6. These two were merged into a single class in the seven-solution model. In the seven-solution model, four classes represented different levels of general usage time while three classes represented high usage time in a specific content. In the six-solution model, class 5 and class 7 of the seven-solution model which showed meaningfully different usage patterns were merged into a single class. Thus, the model with seven profiles was selected and further analyzed. Seven profiles consisted of 147, 1,736, 295, 153, 113, 473, and 67 adolescents each, and its descriptive statistics are presented in Table 4.

**Table 4:** Usage time of Internet, Internet game by PC, and smartphone (h).

	**1**	**2**	**3**	**4**	**5**	**6**	**7**	**Mean**
*N* (%)	147 (4.93)	1,736 (58.18)	295 (9.89)	153 (5.13)	113 (3.79)	473 (15.85)	67 (2.25)	
Internet game by PC	0.29	0.26	0.33	4.97	2.59	2.27	5.34	1.08
Internet by PC	0.68	0.75	4.43	1.48	4.53	1.30	5.19	1.51
Mobile game by smartphone	4.93	1.23	2.00	2.15	2.55	1.75	3.62	1.76
Smartphone	3.70	1.67	3.32	2.43	3.01	2.09	3.67	2.13
Name of profile	“Smartphone user”	“Casual user”	“Internet user”	“PC internet gamer”	“Heavy user”	“Moderate user”	“Excessive user”	

Similar to previous research, four profiles showed generalized usage patterns with different intensity while the other three profiles showed very high usage time in a single or specific context. The first profile (4.93%) displayed the longest time spent on mobile games and using a smartphone, but spent the lowest time on games and the Internet using PC. This profile was named “Smartphone User.” The second profile (58.18%) was named as “Casual User” with short usage time on all four measures. The third profile (9.89%) showed a high usage time on the Internet using both PC and smartphone, very little usage of the PC Internet game, and average usage of mobile game. This profile was named “Internet User.” The fourth profile (5.13%) was named “PC Internet Gamer” with a very high usage time on the PC Internet game and average usage time on other three measures. The fifth profile (3.79%), named “Heavy User,” displayed a high usage time on all four measures. The sixth profile (15.85%) displayed an average usage time on all four measures and was named “Moderate User.” The last profile (2.25%) showed the highest usage time on the Internet and Internet game using PC, and very high usage in both mobile game and smartphone. This profile was named “Excessive User.”

### Validation of Profiles

#### Sex as a Predictor

After the identification of seven meaningful profiles of the Internet and Internet game usage time, the association between sex and the profiles was examined. In Table 5, the odd ratio and statistical significance of being classified into other profiles against the “Casual User” profile are shown. The results indicated that being a male means a higher chance of being classified into the “PC Internet Gamer” [OR = 6.54, *p* < 0.001], “Heavy User” [OR = 2.72, *p* = 0.006], and “Moderate User” [OR = 4.94, *p* < 0.001] profiles compared to the “Casual User” profile. Also, males were more likely to be classified as “Excessive User” [OR = 2.21, *p* = 0.051] than “Causal User” with a marginal significance.

**Table 5:** Male as a predictor of Internet and Internet game usage profile.

**Variable**	**Male as a predictor**	
	**Odd ratio**	***p***
**Reference as “casual user”**		
vs. “Smartphone user”	0.36	<0.001
vs. “Internet user”	0.56	<0.001
vs. “PC internet gamer”	6.54	<0.001
vs. “Heavy user”	2.72	<0.01
vs. “Moderate user”	4.94	<0.001
vs. “Excessive user”	2.21	0.051

#### Differences in Problematic Game Usage and Neuroticism

The differences in problematic game usage among seven profiles were tested using one-way ANCOVA after controlling for the effect of sex and school level. The results are presented in Tables 6, 7. The main effect of profile membership was significant (*F* = 44.861, *p* < 0.001). The *post-hoc* test indicated that the scores of “Casual User” and “Internet User” were the smallest and that those of “Smartphone user” and “Moderate User” were lower than those of “PC Internet Gamer,” “Heavy User,” and “Excessive user” profiles. The scores of “Excessive User” were higher than those of “Heavy User” and “PC Internet Gamer” (*p* < 0.05).

**Table 6:** ANCOVA results comparing problematic game usage among profiles.

	**SS**	***df***	**MS**	***F***	**η^2^**
Profile membership	25,778.639	6	4,296.440	44.861^***^	0.086

*** *p < 0.001*.

**Table 7:** ANCOVA result comparing problematic game usage among profiles (mean difference).

	**Causal user**	**Internet user**	**Moderate user**	**Smartphone user**	**PC Internet gamer**	**Heavy user**	**Excessive user**
Internet user	−0.539	–					
Moderate user	−4.201^*^	−3.662^*^	–				
Smartphone user	−5.654^*^	−5.114^*^	−1.453	–			
PC internet gamer	−7.771^*^	−7.232^*^	−3.570^*^	−2.118	–		
Heavy user	−7.428^*^	−6.889^*^	−3.227^*^	−1.775	0.343	–	
Excessive user	−12.913^*^	−12.373^*^	−8.712^*^	−7.259^*^	−5.142^*^	−5.484^*^	–
Mean	30.674	31.214	34.875	36.328	38.446	38.103	43.587

* *p < 0.05*.

In neuroticism, the main effect was significant (*F* = 8.169, *p* < 0.001). The *post-hoc* test indicated that the neuroticism scores of “Excessive User,” “Internet User,” and “Smartphone User” were significantly higher compared to the scores of “Casual User” and “Moderate User” (*p* < 0.05). The results are presented in Tables 8, 9.

**Table 8:** ANCOVA result comparing neuroticism among profiles.

	**SS**	***df***	**MS**	***F***	**η^2^**
Profile membership	124.646	6	20.744	8.169^***^	0.016

*** *p < 0.001*.

**Table 9:** ANCOVA result comparing neuroticism among profiles (mean difference).

	**Causal user**	**Internet user**	**Moderate user**	**Smartphone user**	**PC Internet gamer**	**Heavy user**	**Excessive user**
Internet user	−0.467^*^	–					
Moderate user	−0.074	0.393^*^	–				
Smartphone user	−0.574^*^	−0.107	−0.500^*^	–			
PC internet gamer	−0.272	0.195	−0.198	0.301	–		
Heavy user	0.173	0.294	−0.099	0.401	0.099	–	
Excessive user	−0.828^*^	−0.362	−0.754^*^	−0.255	−0.556	−0.655	–
Mean	4.912	5.379	4.986	5.486	5.184	5.085	5.741

* *p < 0.05*.

## Discussion

With the heightened risk of addictive behaviors related to the Internet and Internet gaming among adolescents due to the COVID-19 pandemic, the present study investigated the latent profiles based on the Internet and Internet game usage time. Using LPA, seven profiles were identified and their associations with problematic game usage, sex, and neuroticism were examined. The majority (74.03%) of the participants had “Casual User” and “Moderate User” profiles, with below average or average time spent on all four areas (Internet on PC; Internet game on PC; Internet on smartphone; mobile game on smartphone). “Heavy User” (3.79%) and “Excessive User” (2.25%) profiles exhibited high or very high usage time in all four areas. “PC Internet Gamer” (5.13%), “Smartphone User” (4.93%), and “Internet User” (9.89%) profiles had a very high usage time for only a particular content. The proportion of the profiles with the potential risk of developing IGD was 6.04%. The proportion is comparable to the global IGD prevalence of 1.96% (58) but lower than the IGD prevalence of adolescents reported in Germany [14.3%; (59)] and China [17%; (46)].

### Internet Addiction and Internet Game Disorder

Among the seven identified profiles, only the “Internet User” profile reported high Internet usage time. Compared to the “Internet User” profile, all other profiles except “Casual User” profile showed equal or greater usage time in PC Internet game, mobile game, or both. In fact, when comparing profiles with higher gaming usage to profiles with lower gaming usage, profiles with higher gaming usage scored higher on problematic game usage. In addition, profiles with higher Internet usage scored higher in neuroticism compared to profiles with lower Internet usage. This result partly supports the argument of Griffiths and Pontes (29) that the Internet addiction disorder and GD should be considered as different concepts. The study from Király et al. (31) showed similar results where problematic online gaming was associated with a preference for online gaming only and not for any other online activities, and the association was much stronger for males. Similarly, our results indicated that four out of five profiles related to heavy gaming had a higher proportion of males. In brief, our study adds to a trend of studies that support that the Internet addiction and Internet game addiction are different phenomena and reprove the lingering tendency to use these two terms interchangeably. In South Korea, many preventive and intervention programs targeting Internet addiction and Internet gaming addiction for adolescents still do not distinguish the two phenomena and rather define Internet gaming addiction as a subtype of Internet addiction (60–64).

### Female Adolescent Gamers

Problematic gaming or gaming addiction have been largely associated with being male (65, 66), and even a recent IGD prevalence study reported a higher ratio of IGD prevalence in male adolescents (52). However, the results of the present study suggest that more attention may be warranted for female adolescent gamers. As expected, the “PC Internet Gamer” profile comprised a very high male ratio, and three other gaming profiles (“Moderate user,” “Heavy User,” “Excessive User”) also had a high male ratio.

However, the “Smartphone User” profile had a much higher ratio of females. People included in this profile spent a great amount of time on a smartphone (8.63 h), and more than half of their smartphone usage was for gaming (4.93 h). Moreover, problematic game usage scores of the “Smartphone User” profile were significantly higher than those of “Causal User” and “Internet User” and were not significantly different from that of “Moderate user,” “Heavy User,” and “PC Internet Gamer” profiles, and lower compared to “Excessive User” profiles.

The females in the “Smartphone User” profile may not be classified as smartphone addicts on the outside, and interventions may target smartphone addiction only. However, these female smartphone users are likely to engage in problematic gaming. Studies have suggested that the negative consequences of gaming may be different for adolescent gamers by sex. For instance, the reward and lose processing within IGD activates in the opposite direction in males and females (66). In addition, recent brain studies revealed that there are differences in brain activity and features regarding Internet gaming (67, 68) in females that they could be more vulnerable to IGD (65). In the present study, the “Smartphone User” profile was one of three profiles (“Internet User” and “Excessive User”) with higher neuroticism scores. In accordance with the result of the present study and recent studies about female gamers, King and Potenza (69) argued that more attention about female gamers is required in both clinical and research fields as only limited numbers of studies have been conducted (69). However, because this study's findings were based on cross-sectional data, the directionality of relationships cannot be assumed, and confounding is a possibility.

### Mobile Gamer vs. PC Internet Gamer

The game industry and gaming culture have gone through drastic changes, especially in the realm of Internet gaming. Unlike in the past when the Internet was more common with few Internet gamers (31), today almost everyone plays games and increasingly more people are playing Internet games on smartphones than on PCs (34). In 2020, 58.5% of South Korean adolescent gamers played on PCs while 82.4% of them played on smartphones (16). In the present study, the average time spent on playing mobile games (1.76 h) was higher than the average time spent on gaming (1.08 h) and Internet surfing (1.51 h) using PCs.

Although the Internet gaming trend has moved from PCs to smartphones in South Korea, the Korean government policies, research studies, and intervention programs still place more focus on PC Internet games. Moreover, many gaming addiction studies in South Korea still apply the theoretical base and measures from the study of Young et al. (1999), which was groundbreaking back then but is now more than 20 years old (70).

More recently, the interaction of the person–affect–cognition–execution model (71) describes the addiction process as the interaction of context, reaction, and consequence of behaviors. Considering the difference between a mobile game and a PC Internet game, the context, purpose, and consequences of playing a mobile game and a PC Internet game may take different paths and necessitate different interventions. The bigger problem is that most Internet game studies and intervention programs in South Korea utilize Internet addiction measures rather than Internet game addiction scales (70), and they are not based on recent behavioral addiction models. Internet gaming research in South Korea must be updated with the changing industry and cultural trends.

## Limitations

There are three major limitations in the present study. First, there are measurement issues within the study. All measures were self-report measures, and the participants were asked to recall their time spent on the Internet and Internet gaming retrospectively. Compared to measuring the usage time directly by using the time-keeping application or software, self-reported and retrospective information may not be as reliable. Also, social desirability might have influenced the reported time spent on gaming. In South Korea, despite its prevalence, gaming is stigmatized within the society and often blamed as the cause of criminal acts as serious as murder incidents (70). Lastly, because the sampling procedure was not probability sampling, the representativeness of the sample may be undermined. However, the sample was based on the Statistical Yearbook of Education of the Ministry of Education in South Korea, which has sampling guidelines that aim for acceptable representativeness.

Second, the survey sought to measure problematic game usage but did not include Internet addiction-related scales. The results from the present study suggest that Internet addiction might be different from Internet gaming addiction in terms of problematic game usage and the sex ratio. However, a direct comparison using an Internet addiction scale and a problematic game usage scale may prove to be useful. In addition, ANCOVA results of the present study do not convey any directionality and causality between problematic game usage, sex, and neuroticism.

Lastly, the present study sought to investigate the Internet and gaming profiles of adolescents in South Korea during the COVID-19 pandemic. As different literatures suggest, many scholars warned about a heightened risk for developing behavior addiction such as Internet addiction and IGD. However, the current study did not provide any information or analysis that compares the Internet and gaming behaviors before the pandemic and during the pandemic. Thus, no implications can be made regarding the influence of the pandemic on these behaviors.

## Conclusion

This study provides an analysis of Internet usage and Internet gaming behavior of a nationally representative sample in South Korea during the COVID-19 pandemic. The results from the present study partly support the idea that the IGD is a distinct disorder from Internet addiction. In addition, the findings support the notion that mobile gamers are different from PC Internet gamers. Lastly, this study provides a substantial support for creating a greater attention on female gamers in practice and research.

## Data Availability Statement

The data analyzed in this study is subject to the following licenses/restrictions: The datasets analyzed for this study cannot be made publicly available. The dataset is from the national survey by the National Youth Policy Institute in South Korea in 2020, and the authors do not have the right to release the dataset. Requests to access these datasets should be directed to Dongil Kim, dikimedu@snu.ac.kr.

## Ethics Statement

Ethical approval was not provided for this study on human participants because the present study used anonymized secondary data from the survey from the National Youth Policy Institute in South Korea. Written informed consent for participation was not required for this study in accordance with the national legislation and the institutional requirements.

## Author Contributions

DK: writing of the first draft and final draft of the manuscript, acquisition of data, interpretation of analysis, concept and design of the research, and final approval for publication. JL: writing of the first draft and final draft of the manuscript, analysis and interpretation of data, concept and design of the research, and final approval for publication. JN: writing of the final draft of the manuscript, interpretation of data, and final approval for publication.

## Conflict of Interest

The authors declare that the research was conducted in the absence of any commercial or financial relationships that could be construed as a potential conflict of interest.

## Publisher's Note

All claims expressed in this article are solely those of the authors and do not necessarily represent those of their affiliated organizations, or those of the publisher, the editors and the reviewers. Any product that may be evaluated in this article, or claim that may be made by its manufacturer, is not guaranteed or endorsed by the publisher.
